# Association Between Dual-Task Gait and Cognitive Function in Older Adults

**DOI:** 10.1093/geroni/igab046.617

**Published:** 2021-12-17

**Authors:** Anisha Suri, Jessie VanSwearingen, Mark Redfern, Ervin Sejdic, Andrea Rosso

**Affiliations:** 1 University of Pittsburgh, Pittsburgh, Pennsylvania, United States; 2 School of Health and Rehabilitation Sciences, Pittsburgh, Pennsylvania, United States; 3 Swanson School of Engineering, University of Pittsburgh, Pittsburgh, Pennsylvania, United States

## Abstract

Community mobility involves walking with physical and cognitive challenges. In older adults (N=116; results here from initial analyses: N=29, Age=75±5 years, 51% females), we assessed gait speed and smoothness (harmonic-ratio) while walking on even and uneven surfaces, with or without an alternate alphabeting dual-task (ABC). ANOVA assessed surface and dual-task effects; Pearson correlations compared gait with global cognition and executive function composite z-scores. The four conditions (even, uneven, even-ABC and uneven-ABC) affected speed(m/s) (0.97±0.14 vs 0.90±0.15 vs 0.83±0.17 vs 0.79±0.16). Smoothness (2.19±0.48 vs 1.89±0.38 vs 1.92±0.53 vs 1.7±0.43) was affected by only surface (controlled for speed). Greater speed was associated with better global cognition(ρ=0.47 to 0.49, p<0.05) for all conditions and with better executive function for even-ABC(ρ=0.39, p=0.04) and uneven-ABC(ρ=0.40, p=0.03). Executive function was associated with smoothness during even(ρp=-0.42, p=0.03) and uneven(ρp=-0.39, p=0.04) walking. Type of walking challenge differentially affects gait quality and associations with cognitive function.

